# Intestinal microbiota in pediatric patients with end stage renal disease: a Midwest Pediatric Nephrology Consortium study

**DOI:** 10.1186/s40168-016-0195-9

**Published:** 2016-09-17

**Authors:** Janice Crespo-Salgado, V. Matti Vehaskari, Tyrus Stewart, Michael Ferris, Qiang Zhang, Guangdi Wang, Eugene E. Blanchard, Christopher M. Taylor, Mahmoud Kallash, Larry A. Greenbaum, Diego H. Aviles

**Affiliations:** 1 Children’s Hospital, New Orleans, LA USA; 2 Louisiana State University Health Sciences Center, New Orleans, LA USA; 3 Xavier University of Louisiana, New Orleans, LA USA; 4 University at Buffalo, Buffalo, NY USA; 5 Emory University and Children’s Healthcare of Atlanta, Atlanta, GA USA

**Keywords:** End-stage renal disease, Children, Intestinal microbiota, Uremic toxins, Inflammation, Pyrosequencing

## Abstract

**Background:**

End-stage renal disease (ESRD) is associated with uremia and increased systemic inflammation. Alteration of the intestinal microbiota may facilitate translocation of endotoxins into the systemic circulation leading to inflammation. We hypothesized that children with ESRD have an altered intestinal microbiota and increased serum levels of bacterially derived uremic toxins.

**Methods:**

Four groups of subjects were recruited: peritoneal dialysis (PD), hemodialysis (HD), post-kidney transplant and healthy controls. Stool bacterial composition was assessed by pyrosequencing analysis of 16S rRNA genes. Serum levels of C-reactive protein (CRP), D-lactate, p-cresyl sulfate and indoxyl sulfate were measured.

**Results:**

Compared to controls, the relative abundance of Firmicutes (*P* = 0.0228) and Actinobacteria (*P* = 0.0040) was decreased in PD patients. The relative abundance of Bacteroidetes was increased in HD patients (*P* = 0.0462). Compared to HD patients the relative abundance of Proteobacteria (*P* = 0.0233) was increased in PD patients. At the family level, Enterobacteriaceae was significantly increased in PD patients (*P* = 0.0020) compared to controls; whereas, Bifidobacteria showed a significant decrease in PD and transplant patients (*P* = 0.0020) compared to control. Alpha diversity was decreased in PD patients and kidney transplant using both phylogenetic and non-phylogenetic diversity measures (*P* = 0.0031 and 0.0003, respectively), while beta diversity showed significant separation (*R* statistic = 0.2656, *P* = 0.010) between PD patients and controls. ESRD patients had increased serum levels of p-cresyl sulfate and indoxyl sulfate (*P* < 0.0001 and *P* < 0.0001, respectively). The data suggests that no significant correlation exists between the alpha diversity of the intestinal microbiota and CRP, D-lactate, or uremic toxins. Oral iron supplementation results in expansion of the phylum Proteobacteria.

**Conclusions:**

Children with ESRD have altered intestinal microbiota and increased bacterially derived serum uremic toxins.

## Background

The intestinal microbiota is the largest symbiotic ecosystem in the human body. The gastrointestinal tract (GI) is colonized by trillions of bacteria. In healthy adults, almost 90 % of the identified fecal microbiota can be classified into two predominant phyla, Bacteroidetes and Firmicutes. The intestinal microbiota is highly variable during the first years of life, reaching an adult-like composition by 3 years and remains relatively stable [1]. Changes in the composition of the intestinal microbiota have been implicated in the pathogenesis of multiple diseases in children and adults [2, 3].

Patients with end stage renal disease (ESRD) have an increase in cardiovascular disease which cannot be explained by traditional risk factors alone. There is evidence that uremia in ESRD disrupts the intestinal barrier structure, specifically the tight junctions, and impairs intestinal motility [4–7]. These changes may facilitate the translocation of endotoxins and bacterial metabolites into the systemic circulation leading to systemic micro-inflammation [8, 9] and increased cardiovascular disease [10–13]. Blood markers such as D-lactate and endotoxin lipopolysaccharide (LPS), which are metabolic products or components of intestinal bacteria, have been used as indicators of intestinal permeability [14]. Evaluation of intestinal wall integrity is a challenge in patients with reduced renal residual function, because it depends on renal excretion of the orally ingested molecules.

Several factors may influence the intestinal microbiota of ESRD patients. The dietary restriction of potassium may inadvertently cause a reduction in the insoluble fiber intake of these patients [15]. This may ultimately lead to a reduction of normal gut flora which ferment fiber to produce short chain fatty acids (SCFA) which act as a key energy source for colorectal tissues and symbiotic microbes [16]. Further, studies in adults demonstrate that uremia results in a shift in the intestinal microbiota, favoring bacteria capable of surviving in the new biochemical environment [17]. The metabolites produced by this altered colonic flora result in the accumulation of uremic toxins such as indoxyl sulfate and p-cresyl sulfate [18]. Eighteen bacterial families with 4-hydroxyphenylacetate decarboxylase (4-Hpd) and tryptophanase are responsible for the formation of p-cresyl sulfate and indoxyl sulfate, respectively [15, 19]. In addition, recent studies have demonstrated that uremia may also increase ammonia production leading to increased luminal pH and resulting in mucosal damage and irritation [15]. These uremic toxins, which are normally excreted by the kidney via tubular secretion, are poorly cleared by dialysis [20–22]. Several investigators have reported an association between elevated levels of p-cresyl sulfate and indoxyl sulfate with an increased risk of cardiovascular disease and other adverse effects in adults with ESRD [23, 24].

The impact of uremia on the intestinal microbiota of pediatric patients with ESRD is unknown. It is also unclear if renal transplantation has an effect on the composition of the intestinal microbiota, uremic toxin production, or systemic inflammation. Hence, our primary aim was to study the intestinal microbiota of pediatric patients with ESRD and after kidney transplantation. Our secondary aim was to evaluate the correlation between altered intestinal microbiota, D-lactate, C-reactive protein (CRP), and the presence of bacterially derived uremic toxins such as p-cresyl sulfate and indoxyl sulfate.

## Methods

### Study population

This is a cross sectional study comparing four groups of pediatric patients (2 to 18 years of age). Thirty-nine patients were enrolled: eight patients on hemodialysis (HD), eight patients on peritoneal dialysis (PD), ten patients with a kidney transplant and 13 age-matched healthy controls. Participants were recruited from participating centers of the Midwest Pediatric Nephrology Consortium (Children’s Hospital of New Orleans, Women and Children’s Hospital of Buffalo, and Children’s Healthcare of Atlanta) from June 2014 to March 2015. Patient demographic and clinical characteristics are shown in Table 1.

**Table 1:** Cohort characteristics

	Control	ESRD-HD	ESRD-PD	Transplant
*N* (blood/stool)	13/11	8/7	8/7	10/9
Sex (male:female)	6:7	5:3	4:4	10:0
Age (years)	9.5 (3–16)	13.6 (8–17)	11.9 (3-17)	13.2 (2–18)
BMI (kg/m2)	23.15 (14.9–53)	22.1 (16.6–28)	17.8 (14.8–23.4)	18.8 (15.3–23.8)
Time on dialysis (months)	N/A	23.3 (2–77)	21.5 (3–49)	N/A
Kt/v	N/A	1.8 (1.22–2.7)	2.2 (1.89–2.5)	N/A
Time from transplant (month)	N/A	N/A	N/A	43.1 (10–110)
BUN (mg/dl)	13.54 (8–19)	51.00 (33–125)	41.38 (14–65)	17.78 (9–26)
Serum creatinine (mg/dl)	0.37 (0.10–0.70)	8.58 (0.90–18.10)	11.70 (7.40–19.00)	0.99 (0.50–1.60)
eGFR	118 (85-139)	<15	<15	78 (61–108)

*ESRD-HD* end-stage renal disease on hemodialysis, *ESRD-PD* end-stage renal disease on peritoneal dialysis, *N/A* not available, *N* number of samples, *Kt/v* dialysis dose, *BUN* blood urea nitrogen, *eGFR* estimated glomerular filtration rate

We excluded patients with history of diabetes, pancreatitis, cirrhosis, inflammatory bowel disease, diarrhea, severe constipation, or use of antibiotics within 1 month. Additional exclusion criteria included kidney transplant recipients with an estimated glomerular filtration rate (eGFR) of less than 60 ml/min/1.73 m2, acute rejection or transplant within the last 6 months. Written informed consent was obtained from a legal guardian or the patient if 18 years old, and assent was obtained in patients older than 7 years. The study was approved by the Louisiana State University Health Sciences Center Institutional Review Board (IRB # 8554).

### Stool sample collection

One stool sample (1–2 g) was collected from each subject. We provided instructions describing how to collect stool specimens, place them in nucleic acid preservative, and deliver them to our Research Center within 24 to 48 h after collection. Stool specimens were collected in Assay Assure (Sierra Molecular, Incline Village, NV), which does not require refrigeration. DNA was isolated from the stool samples within 48 h of receipt and stored at −20 °C.

### 16S rRNA sequencing

Stool DNA was isolated using a QIAamp Fast DNA Stool Mini kit (Qiagen, Germantown, Maryland) modified to include a sonication step. Sequencing and bioinformatics were performed by the Louisiana State University Microbial Genomics Resource Center. The 16S ribosomal DNA hypervariable regions V3 and V4 were PCR-amplified using primers V3F CCTACGGGAGGCAGCAG and V4R GACTACHVGGGTWTCTAAT with Illumina adaptors and molecular barcodes as described in Kozich et al. [25] to produce 430 base pair (bp) amplicons. Samples were sequenced on an Illumina MiSeq (Illumina, San Diego, CA) using a V3 sequencing kit (300 bp paired end reads).

The forward and reverse-read files were processed through the UPARSE pipeline (drive5, Tiburon, California) [26]. Forward and reverse reads were merged with a merge truncation quality of 2 as recommended by Edgar for Illumina data. Merged amplicons were filtered using a filter truncation quality of ten and resulting amplicons were truncated to a length of 400 bp. Singleton reads (reads that appeared only once across all samples) were removed from the resulting filtered reads and remaining reads were clustered into operational taxonomic units (OTUs) at 97 % similarity. Chimeric OTUs were removed as identified by UCHIME drive5 run against a gold standard reference database (ChimeraSlayer reference database) of nonchimeric sequences.

Finally, the original filtered reads (before dereplication) were mapped to the OTUs using USEARCH drive5 at 97 % identity. QIIME 1.8 (open source, www.qiime.org) was used to pick and align a representative set. The Ribosomal Database Project classifier was used to assign a taxonomic classification to each read in the representative set and a phylogenetic tree was constructed from the representative sequences. Among samples, the minimum read count after filtering was 7371 reads and the median read count after filtering was 13,226 reads. The OTUs were assigned to the lowest possible taxonomic level. Bacterial alpha diversity among groups was calculated using phylogenetic whole tree and Chao 1 within QIIME. The bacterial beta diversity was assessed using the principal coordinates analysis (PCoA) of weighted UniFrac distances within QIIME.

### Blood sample collection

Blood specimen was drawn as part of routine laboratory sampling. For HD patients, blood was taken immediately prior to the midweek dialysis session. Using a 5-ml serum separator tube (SST) with polymer gel and clot activator, 5 ml of venous blood was collected. Following standard separation procedure, serum was isolated and stored at −80 °C. Renal function was assessed by measuring blood urea nitrogen (BUN) and creatinine then calculating the estimated glomerular filtration rate (eGFR) using the modified Schwartz formula [27] (Table 1).

### Bacterially derived uremic toxins

Serum p-cresyl sulfate and indoxyl sulfate were measured by high performance liquid chromatography (HPLC) as described by de Loor et al. [28]. Briefly, two aliquots of 200 μL of serum were added 20 μL of 0.50 mM 1-naphthalenesulfonic acid (internal standard) and vortex-mixed with 250 μL of 0.24 M sodium octanoate (binding competitor). After incubation at room temperature for 5 min, 2 mL of cold acetone was added to precipitate proteins. Following vortex-mixing and centrifuging at 4°C, 1860×*g* for 20 min, the supernatant was transferred to 12 × 100 mm, GL 14 glass test tubes and 2 mL of dichloromethane was then added. After vortex-mixing and centrifuging at 4°C, 1860×*g* for 10 min, 200 μL of the upper layer was transferred to glass auto-sampler vials, followed by addition of 20 μL 1 M HCl. 15 μL of this final solution was used for HPLC analysis.

A Shimadzu (Columbia, MD) Prominence LC-20AT HPLC system with UV/Fluorescence detector and auto sampler was used for determination of p-cresyl sulfate and indoxyl sulfate. The analytes were resolved by using a reverse phase Sunfire C18 column (150 × 3.0 mm; 3.5 μm particle size; Waters) with a Sunfire C18 guard column (20 × 3.0 mm; 3.5 μm particle size; Waters) at a flow rate of 0.5 ml/min. Mobile phase A was 0.2 % trifluoroacetic acid in Milli-Q water and mobile phase B was 0.2 % trifluoroacetic acid in acetonitrile. The analytical method consisted of a run with gradient elution starting at 8 % phase B for 5 min, followed by a linear increase to 18 % in 15 min, a linear change to 100 % in 5 min, a hold for 3 min and a final linear change back to 8 % phase B in 2 min, a return to the initial condition for 10 min before next injection. Indoxyl sulfate eluted at 13.8 min, *p*-cresyl sulfate at 20.4 min and internal standard at 23.7 min. Column temperature was 25 °C, and autosampler tray temperature was 6 °C. We quantified the analytes by using the analyte to standard peak area ratios on a Shimadzu RF-20A xs fluorescence detector. Detector settings were λex 260 nm/λem 288 nm for *p*-cresyl sulfate and λex 280 nm/λem 390 nm for indoxyl sulfate and internal standard.

### Calibration curves

Calibrators containing indoxyl sulfate and *p*-cresyl sulfate at a final concentration between 1.5 and 400.0 μM were prepared in phosphate buffered saline. Two calibration curves were constructed with a linear response ranging from 1.5 to 50.0 μM (low) and 1.5 to 400.0 μM (high). Regression equations were reported as mean (standard deviation (SD)) from three independent curves for indoxyl sulfate were as follows:

- *y* = 0.06 × (0.00072) + 0.3 (0.11) (high curve)
- *y* = 0.07(0.0018) × ‐ 0.01 (0.0042) (low curve)

The regression equations for *p*-cresyl sulfate were as follows:

- *y* = 0.0083(0.00057) × + 0.02(0.0088) (high curve)
- *y* = 0.0092(0.00036) × + 0.003(0.00086) (low curve)

Overall coefficient of determination (*R*2) was 0.999

### Inflammatory marker and D-lactate measurement

Serum CRP was determined using the human C-Reactive Protein ELISA kit (Sigma-Aldrich, St. Louis, MO) according to manufacturer’s instruction. Serum D-lactate level was determined using the D-lactate colorimetric assay kit (Sigma-Aldrich, St. Louis, MO) according to the manufacturer’s instruction.

### Statistical analysis

Alpha diversity was measured using phylogenetic diversity whole tree and Chao1 within QIIME, using the average of ten iterations in which 6600 sequences were randomly sampled from each specimen. Diversity was compared between PD, HD, post-transplant, and control groups. Statistical differences in bacterial community composition between groups of specimens were assessed using analysis of similarity (ANOSIM) and principal coordinates analysis (PCoA) of weighted UniFrac distances within QIIME [29]. Comparison of multiple groups was performed by the nonparametric Kruskal-Wallis test followed by Dunn’s multiple comparisons test within GraphPad PRISM 6 (Graph Pad Software Inc., San Diego, CA). Correlation analysis was assessed by Spearman nonparametric correlation within PRISM. Box and whisker plots illustrate median (line within the box) and minimum and maximum values (whiskers).

## Results

### Relative abundance of bacterial taxa

**Fig. 1:**
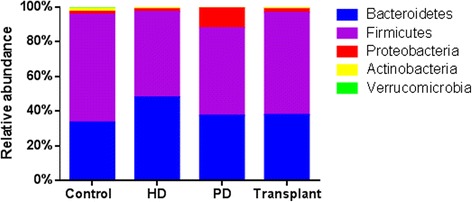
Taxonomic composition of the intestinal microbiota. Percent abundance of bacterial phyla

**Fig. 2:**
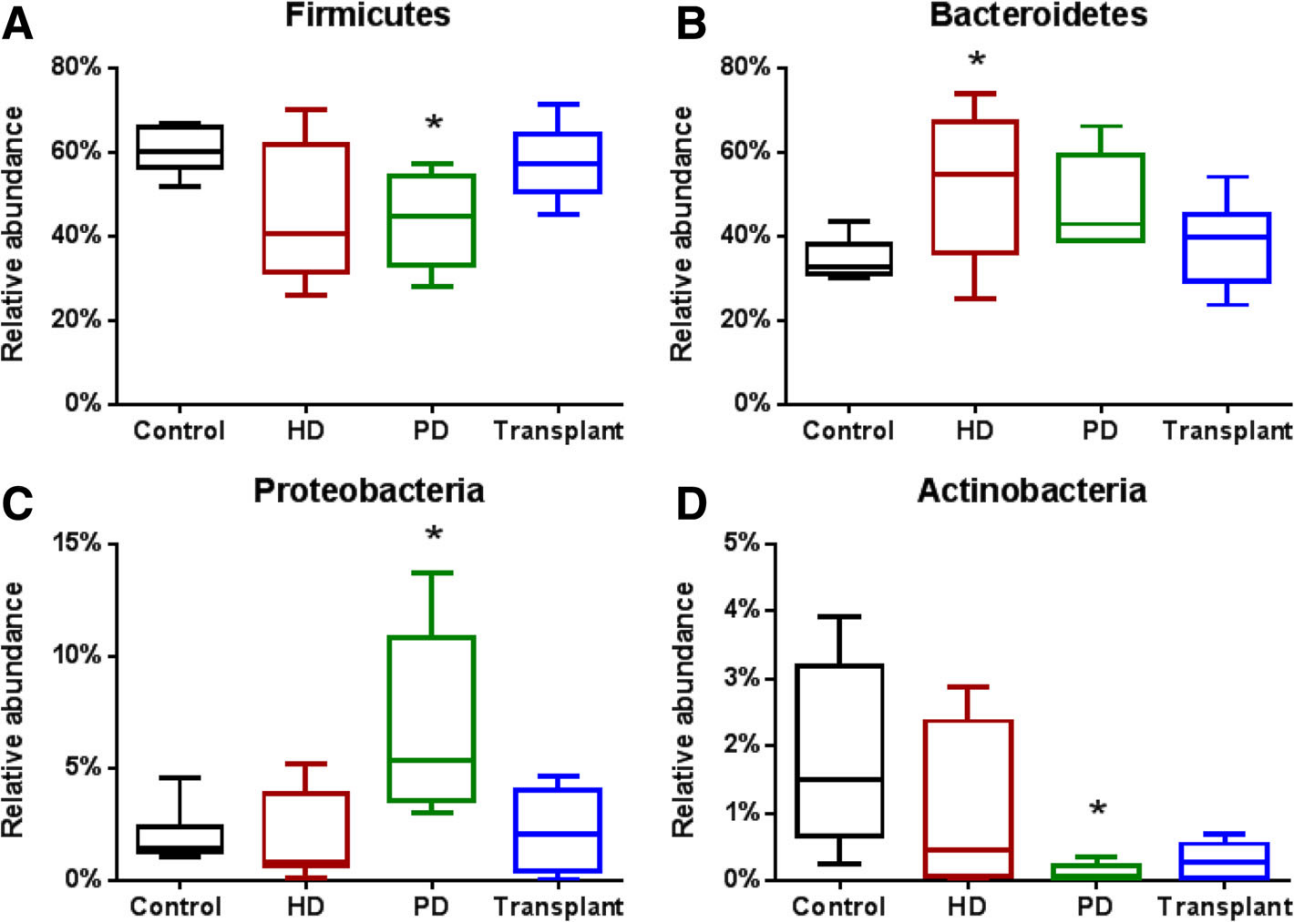
Relative abundance of the four dominant phyla of the intestinal microbiota. **a** Patients on PD showed significantly reduced abundance in Firmicutes (*P* = 0.0228) when compared to healthy control. **b** Bacteroidetes was significantly increased in HD patients (*P* = 0.0462) when compared to control. **c** Proteobacteria was significantly increased in PD patients (*P* = 0.0233) when compared to HD patients. **d** The abundance of Actinobacteria was significantly decreased in PD patients (*P* = 0.004) when compared to control. Kruskal-Wallis test followed by Dunn’s multiple comparisons test. *N* = 6–11 in individual groups. *Box* and *whisker plots* illustrate median (*line* within the *box*) and minimum and maximum values (*whiskers*)

There were notable differences in the relative abundances of intestinal bacteria at the phylum level between patient groups (Fig. 1). The relative abundance of bacteria within the phylum Firmicutes was significantly lower in PD patients (*P* = 0.0228) compared to healthy controls (Fig. 2a). Whereas the relative abundance of the phylum Bacteroidetes was significantly increased in HD patients (*P* = 0.0462) compared to healthy controls (Fig. 2b). There were also significant differences in the relative abundances of phyla across dialysis modalities. The phylum Proteobacteria was significantly increased in patients on PD (*P* = 0.0233) compared to patients on HD (Fig. 2c). Actinobacteria was significantly decreased in PD patients (Fig. 2d) compared to controls (*P* = 0.0040).

### Bacterial diversity

**Fig. 3:**
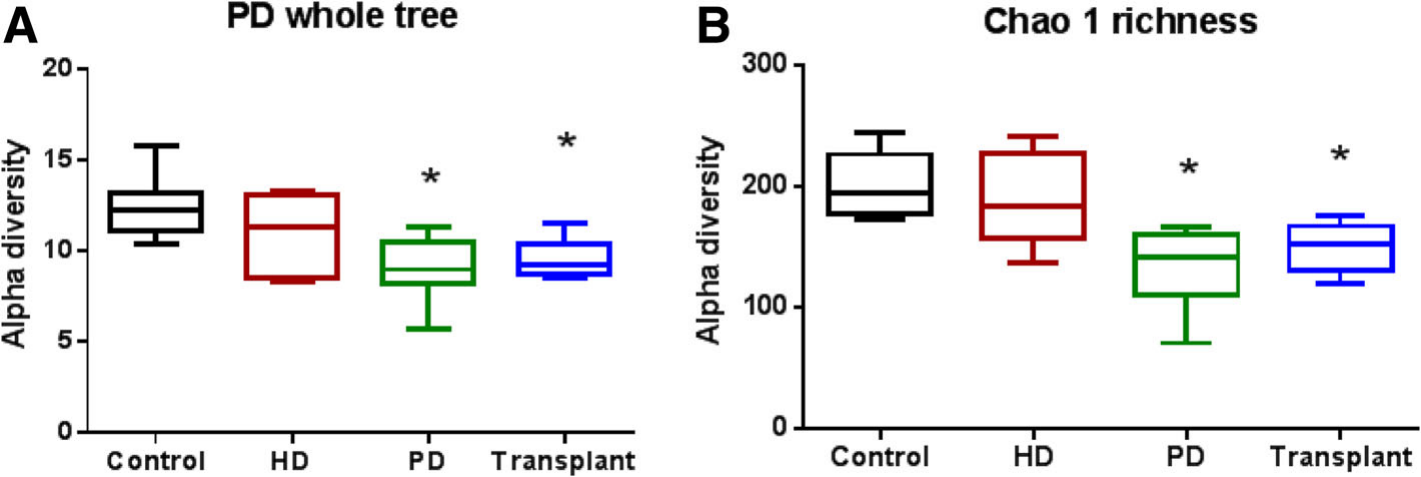
Microbial alpha diversity. Box plots were generated to compare the bacterial richness within groups. Patients with ESRD on PD as well as kidney transplant patients had significantly decreased alpha diversity as demonstrated by different metrics of alpha diversity: (**a**) phylogenetic diversity whole tree (*P* = 0.0031) and (**b**) chao-1 richness estimation (*P* = 0.003). Kruskal-Wallis test followed by Dunn’s multiple comparisons test. *N* = 7–11 in individual groups. *Box* and *whisker plots* illustrate median (*line* within the *box*) and minimum and maximum values (*whiskers*)

Alpha diversity was significantly decreased in patients on PD and in patients with kidney transplants, when compared to patients on HD and controls, using both phylogenetic (Fig. 3a) and non-phylogenetic (Fig. 3b) diversity measures (*P* = 0.0031 and 0.0003, respectively).

### Bacterial community composition

**Fig. 4:**
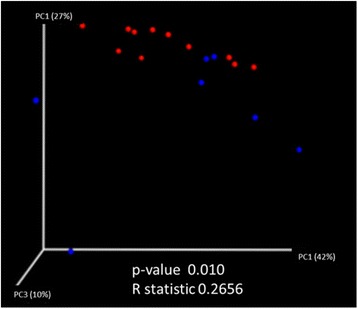
Microbial beta diversity. Principal coordinate analysis (PCoA) of PD patients compared to control. Weighted UniFrac distance showed significant separation between microbial communities in patients with ESRD on PD (*blue dots*) and the healthy control patients (*red dots*) (*R* statistic = 0.2656, *P* = 0.010)

PCoA analysis of weighted UniFrac distances showed significant separation between microbial communities (Fig. 4) of patients with ESRD on PD compared to healthy control group, (*R* statistic = 0.2656, *P* = 0.010, ANOSIM).

### Indole and P-cresol producing taxa

**Fig. 5:**
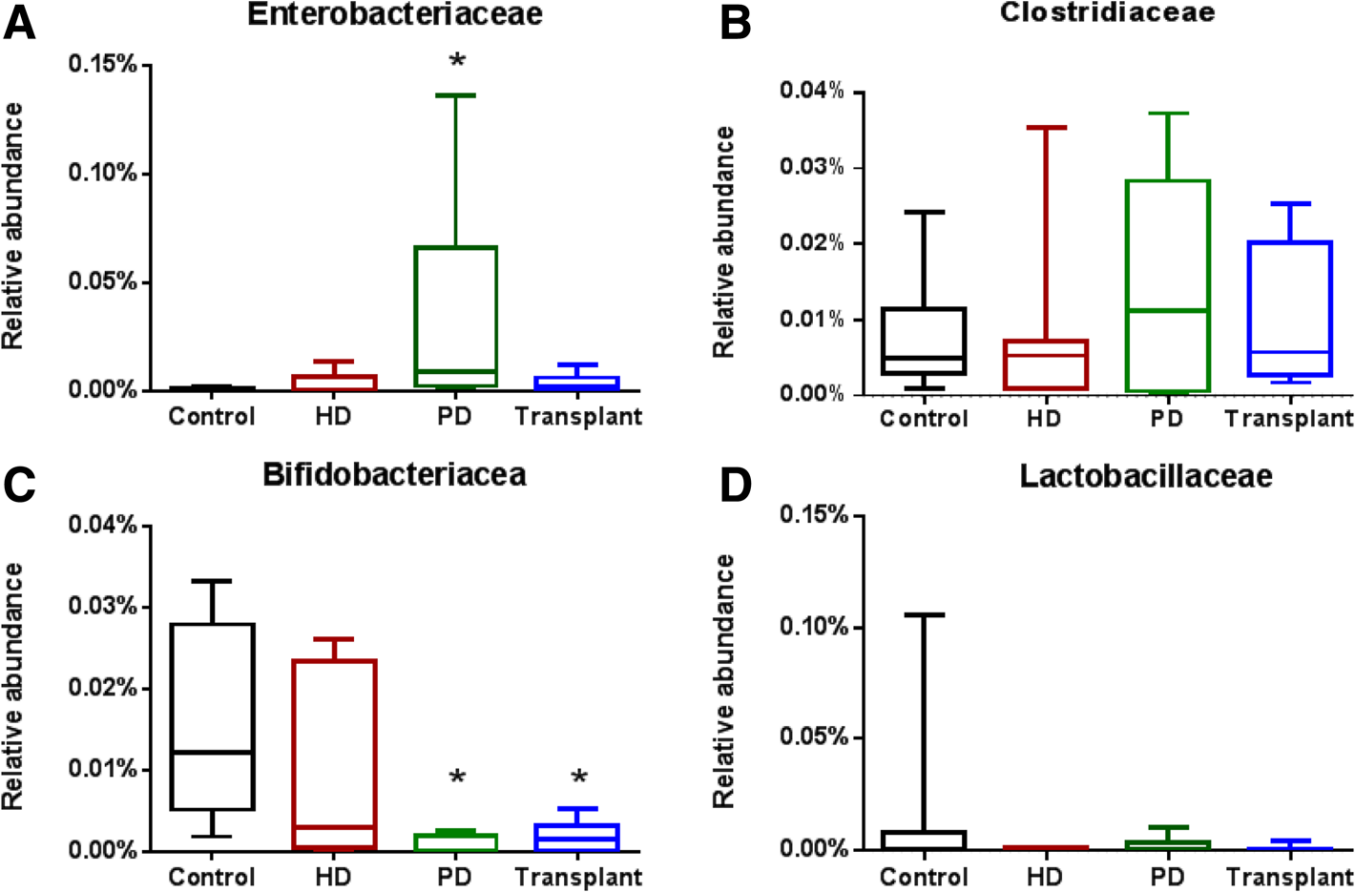
Relative abundance of intestinal microbiota at the family level. **a** Enterobacteriaceae was significantly increased in patients on peritoneal dialysis (*P* = 0.0020). **b** Clostridiaceae showed no significant difference among the groups (*P* = 0.8378). **c** The abundance of Bifidobacteriaceae was significantly decreased in PD and transplant patients (*P* = 0.0020). **d** There were no significant differences in Lactobacillaceae among the groups (*P* = 0.8765). Kruskal-Wallis test followed by Dunn’s multiple comparisons test. *N* = 6–11 in individual groups. *Box* and *whisker plots* illustrate median (*line* within the *box*) and minimum and maximum values (*whiskers*)

Enterobacteriaceae was significantly increased in patients with ESRD who were on PD (Fig. 5a) compared to controls (*P* = 0.0020). We found no significant difference in Clostridiaceae across any patient groups (Fig. 5b). Bifidobacteriaceae was significantly decreased (*P* = 0.0020) in PD and with kidney transplant patients when compared to controls (Fig. 5c). There was no significant difference observed in the Lactobacillaceae family between any of the patient groups (Fig. 5d).

### Serum p-cresyl sulfate and indoxyl sulfate levels

**Fig. 6:**
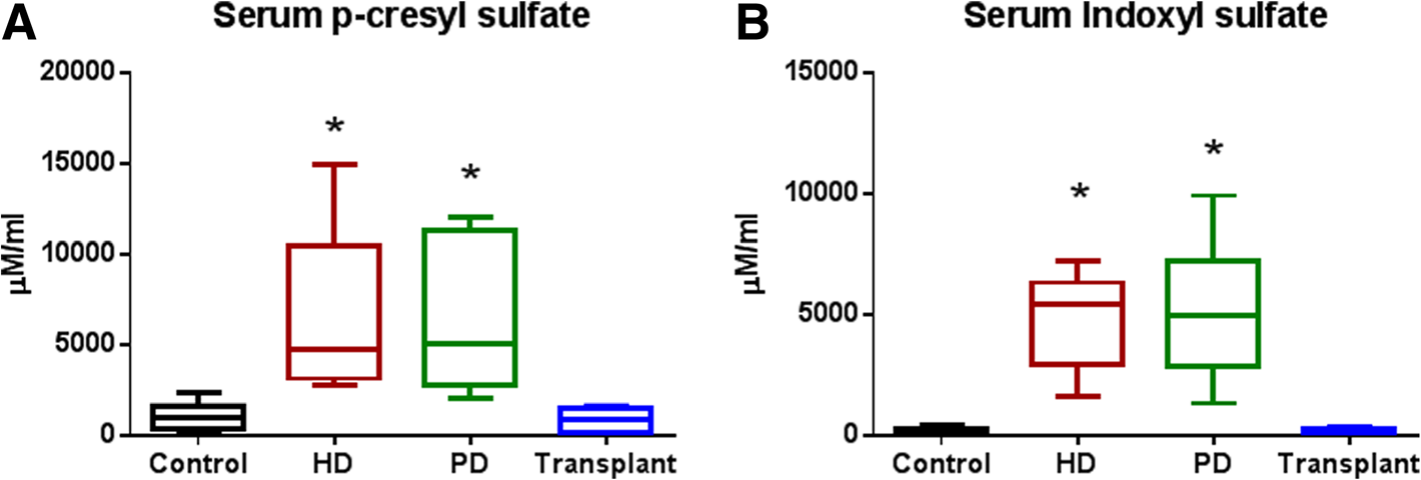
Serum levels of bacterially produced uremic toxins. ESRD patients showed significantly increased levels of (**a**) p-cresyl sulfate *P* < 0.0001 (Kruskal-Wallis test followed by Dunn’s multiple comparisons test.) and (**b**) indoxyl sulfate *P* < 0.0001 (Kruskal-Wallis test followed by Dunn’s multiple comparisons test.). *N* = 7–12 in individual groups. *Box* and *whisker plots* illustrate median (*line* within the *box*) and minimum and maximum values (*whiskers*)

Patients with ESRD, either on HD or PD, had significantly increased levels of the uremic toxins p-cresyl sulfate (*P* < 0.0001) and indoxyl sulfate (*P* < 0.0001) compared to control and transplant patients (Fig. 6a, b, respectively). There was no significant difference between HD and PD patients.

### Serum CRP and D-lactate

**Fig. 7:**
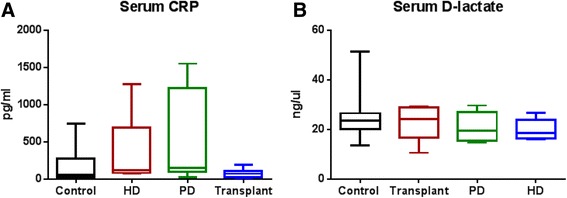
Serum level of CRP and D-lactate. ESRD patients showed no significant differences for (**a**) serum CRP *P* = 0.0590 or (**b**) serum D-lactate *P* = 0.5669 (Kruskal-Wallis test followed by Dunn’s multiple comparisons test.). *N* = 8–12 in individual groups. *Box* and *whisker plots* illustrate median (*line within the box*) and minimum and maximum values (*whiskers*)

There were no significant differences observed for serum CRP or serum d-lactate level among the study groups (Fig. 7a, b).

### Correlation of species richness and serum biomarkers

**Fig. 8:**
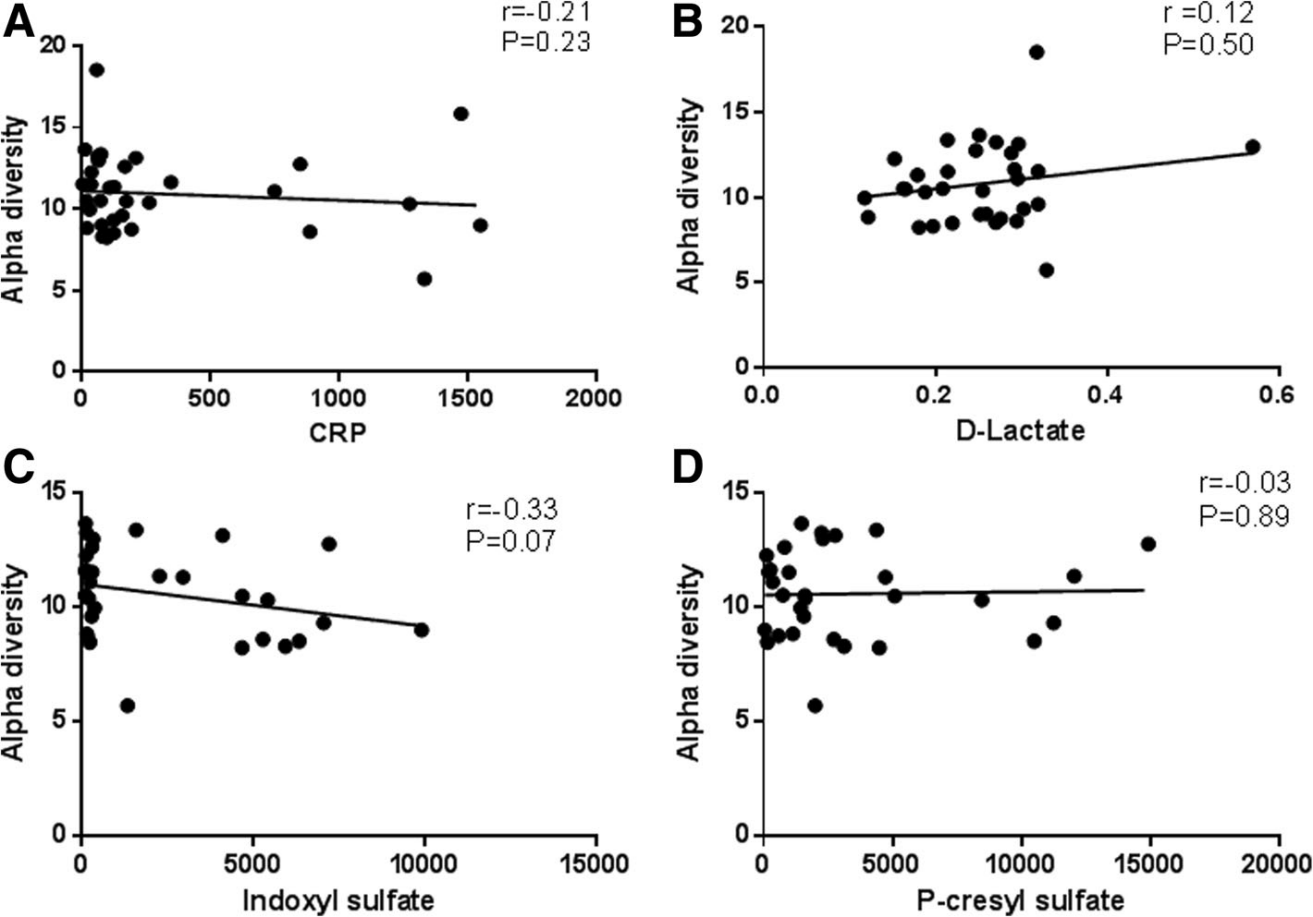
Correlations between alpha diversity and serum biomarkers. Alpha diversity showed no significant correlation between (**a**) serum CRP (*r* = −0.2110, *P* = 0.23), (**b**) serum D-lactate (*r* = 0.12, *P* = 0.50), (**c**) indoxyl sulfate (*r* = −0.12, *P* = 0.07), or (**d**) p-cresyl sulfate (*r* = −0.03, *P* = 0.89). Spearman nonparametric correlation

There was no significant correlation between alpha diversity measures (PD whole tree) and serum CRP, D-lactate, or uremic toxins (Fig. 8).

## Discussion

To our knowledge, this study is the first to assess the composition of the intestinal microbiota of pediatric patients with ESRD or kidney transplants. Recent studies have revealed the fundamental role of the intestinal microbiota in the maintenance of normal human physiology and homeostasis. This complex ecosystem within the gut is highly susceptible to disturbances that can alter the composition of the intestinal microbiota. We demonstrate that children and adolescents with ESRD and kidney transplant exhibit an altered intestinal microbiota. PD patients, as well as those with a kidney transplant, exhibited significantly decreased bacterial diversity (alpha diversity) compared to control. In addition, the composition of bacterial communities (beta diversity) within our PD patients was significantly different when compared to the healthy control group. Serum levels of bacterially derived uremic toxins, p-cresyl and indoxyl sulfate, were significantly increased in ESRD patients.

The GI tract hosts a broad range of bacterial species. It is estimated that between 500 and 1000 species are resident at any given time, but four divisions predominate: Firmicutes, Bacteroidetes, Proteobacteria, and Actinobacteria. Firmicutes constitute ~64 % of the microbiota, whereas Bacteroidetes account for ~23 % of the normal microbiota [30]. In chronic conditions such as type 2 diabetes mellitus and inflammatory bowel disease, patients develop dysbiosis, defined as an abnormal ratio of beneficiary and aggressive bacterial species [31]. We found that patients with ESRD on PD had a decreased relative abundance of the phylum Firmicutes (Fig. 2a), while HD patients exhibited an increase to the phylum Bacteroidetes (Fig. 2b) when compared to healthy control patients. The same pattern of gut dysbiosis was reported in patients with type-2 diabetes [32].

In addition to uremia, several possible mechanisms may account for the observed changes to the intestinal microbiota. Proteobacteria, also known as the iron oxidizing bacteria, are typically present in small amounts in the healthy adult intestinal microbiota [33]. However, our results demonstrate that Proteobacteria were increased in patients on PD (Fig. 2c) when compared to HD patients. This may be due to the oral iron supplementation received by PD patients. Although both ESRD groups receive iron supplementation, the route of delivery differs for the two dialysis modalities. Whereas HD patients receive iron supplementation directly into the bloodstream, PD patients ingest iron supplements. The interaction of orally ingested iron with gut microbes able to metabolize this mineral may account for the increased Proteobacteria levels. Further, Enterobacteriaceae, a family belonging to the phylum Proteobacteria, exhibited a significant increase in abundance among PD patients compared to control patients (Fig. 5a). The Enterobacteriaceae are known to ferment glucose and account for up to 12 % of all peritonitis episodes in patients with ESRD treated with PD [34]. We speculate that the observed increase in Enterobacteriaceae in PD patients may be due to intestinal absorption of glucose from the dialysate. This increase in Enterobacteriaceae may contribute to peritonitis infections observed in PD patients. Future studies are needed to better evaluate these findings.

Patients on PD had a significantly decreased abundance of the phylum Actinobacteria (Fig. 2d). Conversely, Wu et al. demonstrated that increased Actinobacteria was associated with a healthy gut microbiome in children [35]. Bifidobacterium, a family belonging to the phylum Actinobacteria, also exhibited a decreased abundance in PD and kidney transplant patients (Fig. 5c). These organisms might be responsible for a variety of beneficial health effects, including the regulation of intestinal microbial homeostasis and the inhibition of harmful bacteria that colonize or infect the gut mucosa [36]. Bifidobacterium improve the gut mucosal barrier and lower levels of lipopolysaccharide in the intestine [37]. Alterations in the integrity of the intestinal mucosa facilitate the translocation of bacterially derived toxins.

Loss of bacterial diversity has been shown to play a role in the severity of clinical features of infants with necrotizing enterocolitis (NEC) [38] and adult patients with chronic obstructive lung disease (COPD) [39]. This suggests an association between loss of bacterial diversity and disease severity. There was a significant decrease in the number of species (alpha diversity) in our patients on PD and with a kidney transplant, when compared to patients on HD and controls using both phylogenetic (Fig. 3a) and non-phylogenetic diversity measures (Fig. 3b). In future studies, we will address the question of whether the observed alpha diversity in children with ESRD affects the severity or rate of complications. Results of the beta diversity analysis, illustrated by PCoA plot (Fig. 4), showed a significant difference in UniFrac distances between ESRD on PD and control microbial communities. Our results represent an initial insight into the intestinal microbiome patterns in ESRD, which would need to be confirmed in larger cohorts.

Of note, transplant patients exhibited decreased species diversity and decreased abundance of Bifidobacteria (Fig. 3a, b, and Fig. 5c, respectively). Several studies have reported shifts in gut microbiota, including loss of dominant phyla and decreased species diversity, following organ transplantation [40–42]. These alterations may be the result of antibiotic treatment, immunosuppression therapy or a combination of the two. Further studies will be needed to elucidate the mechanism by which these alterations are occurring.

Serum concentrations of p-cresyl sulfate and indoxyl sulfate have been associated with progression of chronic kidney disease [21, 23], increased cardiovascular disease [10–13] and overall mortality in adult patients with chronic kidney disease. Previous studies found that the high protein binding affinity of both bacterially derived uremic toxins markedly limits their removal by dialysis, even with the use of high-flux membranes [43]. Serum levels of p-cresyl sulfate and indoxyl sulfate seem to correlate with loss of residual renal function [44]. Recent animal studies, demonstrated that p-cresyl sulfate and indoxyl sulfate are excreted by renal tubular secretion via organic anion transporters [45]. Similar to adult studies, we found significantly increased levels of p-cresyl sulfate and indoxyl sulfate in our pediatric patients with ESRD. Kidney transplantation results in normalization of serum indoxyl sulfate and p-cresyl sulfate, despite decreased bacterial diversity. These findings suggest that renal clearance via tubular secretion is of paramount importance for maintaining normal serum levels of both toxins. Accumulation of these uremic toxins is considered one of the possible causes of chronic inflammation in patients with ESRD.

Chronic inflammation is a well-established independent risk factor for cardiovascular disease. Patients with ESRD have a high prevalence of micro-inflammation [46–49]. In recent years, several studies have tried to elucidate the etiology for this inflammatory state. The relative roles of residual renal function, dialysis modality, bacterial translocation, intestinal microbiota and the accumulation of endotoxins such as p-cresyl sulfate and indoxyl sulfate in causing the inflammatory milieu of CKD remains unclear. In our study, we assessed inflammation by measuring serum CRP, but did not observe a statistical difference between our groups of patients. There was a trend toward higher serum CRP levels in the patients with ESRD; however, the lack of statistical significance may have been due to the high variation and small sample sizes. Furthermore, we discovered that several of our control patients were overweight or obese, which is a confounding factor for inflammatory markers. Visser et al. demonstrated that higher BMI was associated with increased CRP levels [50].

Evidence suggests that uremia may impair the intestinal barrier function of ESRD patients [4–7]. This “leaky gut” may allow the passage of endotoxins and bacterial metabolites into the systemic circulation contributing to systemic microinflammation [8, 9]. For this study, serum D-lactate was used as an indirect marker of intestinal permeability. There were no significant differences observed between the groups. Although a previous study reports elevated plasma D-lactate levels in ESRD patients, this observation was only seen in conjunction with the presence of bacterial DNA in blood samples [9]. Our study of serum D-lactate level does not include simultaneous analysis of bacterial DNA in blood samples. This suggests that D-lactate alone may not independently indicate the level of intestinal permeability in ESRD patients.

The level of species richness, as measured by PD whole tree, did not show a significant correlation with any of the serum biomarkers used in this study. As stated above, several factors may contribute to the lack of correlation and statistically significant differences among these groups of study. Although some studies have demonstrated the correlation between specific intestinal microbes and biomarkers, to our knowledge this is the first study to attempt to link the overall intestinal species diversity with serum biomarkers in a pediatric population. Further studies of larger cohort populations will be needed to validate the results of this study.

Recent studies suggest that uremic by-products induce toxicity by increasing the production of reactive oxygen species (ROS) through NADPH oxidase (membrane-bound enzyme complex that generate superoxide). ROS production generates the mitogen-activated protein kinase (MAPK)/NF-kB (nuclear factor kB) pathway which leads to the production of pro-inflammatory cytokines, chemokines and adhesion molecules [51, 52]. Measurement of ROS in pediatric ESRD patients is a potential avenue of investigation.

There are limitations in our study. Our sample size was small, and this limited our statistical power. We were unable to control for potential confounding variables such as dietary restrictions or BMI.

## Conclusions

We have demonstrated that pediatric patients with ESRD and kidney transplant have an altered intestinal microbiota. The intestinal microbiota of ESRD patients differs with dialysis modality.

Patients with ESRD have elevated levels of p-cresyl sulfate and indoxyl sulfate.

Renal transplantation results in the normalization of bacterially derived uremic toxins p-cresyl sulfate and indoxyl sulfate; however, renal transplantation does not correct the decreased bacterial diversity.

## Abbreviations

ANOSIM: analysis of similarity
ANOVA: one-way analysis of variance
BUN: blood urea nitrogen
CRP: C-reactive protein
eGFR: estimated glomerular filtration rate
ESRD: end-stage renal disease
GI: gastrointestinal tract
HD: hemodialysis
HPLC: high-performance liquid chromatography
LPS: lipopolysaccharide
MAPK: mitogen-activated protein kinase
NF-kB: nuclear factor kB
OUT: operational taxonomic units
PCoA: principal coordinates analysis
PD: peritoneal dialysis
ROS: reactive oxygen species
SD: standard deviation
SST: serum separator tube
